# Understanding the cellular and molecular alterations in PTSD brains: The necessity of post-mortem brain tissue

**DOI:** 10.1080/20008198.2017.1341824

**Published:** 2017-06-26

**Authors:** Geertje M. de Lange

**Affiliations:** ^a^ NBB-Psy, Netherlands Brain Bank, Netherlands Institute for Neuroscience, Amsterdam, The Netherlands

**Keywords:** Brain Bank, post mortem, PTSD, psychiatry, brain tissue, post-traumatic stress disorder, prospective donor programme

## Abstract

The personal, social and economic burden of post-traumatic stress disorder (PTSD) is high and therapeutic approaches are only partially effective. Therefore, there is an urgent need to understand the cellular and molecular alterations in PTSD brains in order to design more effective treatment strategies. Although brain imaging strategies have considerably improved our understanding of PTSD, these strategies cannot identify molecular and cellular changes. Post-mortem examination of the brain is a crucial strategy to advance our understanding of the underlying neuropathology, neurochemistry and molecular pathways of PTSD. Unfortunately, there is a worldwide serious shortage of human psychiatric brain tissue available for post-mortem research. Therefore, the Netherlands Brain Bank launched a prospective donor programme to recruit brain donors with psychiatric diseases in 2012: Netherlands Brain Bank for Psychiatry (NBB-Psy). NBB-Psy aims to establish a resource of brain tissue of seven psychiatric disorders: post-traumatic stress disorder, major depressive disorder, schizophrenia, bipolar disorder, obsessive-compulsive disorder, autism spectrum disorder, and attention-deficit hyperactivity disorder. Participants of several large and clinically characterized research cohorts of psychiatric patients, including relatives and controls, were asked prospectively to register as brain donors. Registered donors complete medical questionnaires annually. The number of registered donors with a psychiatric disorder at the NBB has risen from 312 (most of which were patients with major depressive disorder) in the year 2010 to 1187 in 2017, of which 146 are PTSD patients. The NBB guarantees worldwide open access to biomaterials and data. Any researcher affiliated with a research institute can apply.

The personal, social and economic burden of post-traumatic stress disorder (PTSD) is high and therapeutic approaches such as prolonged exposure, EMDR, cognitive therapy, and paroxetine and sertraline are only partially effective (Bradley, Greene, Russ, Dutra, & Westen, 2005; Nijdam & Olff, 2016; Schnyder et al., 2015; Steenkamp, Litz, Hoge, & Marmar, 2015). Besides, intense emotions triggered by psychotherapy can be intolerable and can lead to poor compliance (Taylor, 2006) and it remains unknown which clients are more likely to benefit from which treatment method (Olff et al., 2015; Seidler & Wagner, 2006). Therefore, there is an urgent need to understand the cellular and molecular alterations in PTSD brains in order to design more effective treatment strategies. PTSD is a classic example of how brain functions can change as a consequence of an external event. The symptoms of PTSD reflect a persistent, inadequate adaptation of neurobiological systems to the stress of an experienced or witnessed trauma (Lanius & Olff, 2017; Marinova & Maercker, 2015). Neuroimaging studies have demonstrated significant neurobiological changes in PTSD, namely alterations in volume, connectivity and activity, involving the hippocampus, amygdala and the medial frontal cortex (Fragkaki, Thomaes, & Sijbrandij, 2016; Lanius, Frewen, Tursich, Jetly, & McKinnon, 2015; Nutt & Malizia, 2004; Quidé, Witteveen, El-Hage, Veltman, & Olff, 2012). Athough brain imaging strategies have considerably improved our understanding of PTSD, these strategies cannot identify molecular and cellular changes. In addition, animal models of psychiatric disorders have limited validity because they do not reflect the complexity of the human brain and because some symptoms such as suicidality cannot be simulated in animal models. Furthermore, a blueprint of processes in the brain is not likely to be found in peripheral material (e.g. blood), because of the intact blood–brain barrier in psychiatric disorders and the unique properties of brain tissue. Given these limitations, post-mortem examination of the brain is a crucial strategy to advance our understanding of the underlying neuropathology, neurochemistry and molecular pathways of PTSD. And yet, post-mortem studies on PTSD are rare. Literature search only shows two studies in which only nine PTSD brains were examined (Bracha, Garcia-Rill, Mrak, & Skinner, 2005; Su et al., 2008). Bracha et al. (2005) found reduced numbers of locus coeruleus neurons in the brains of three elderly male veterans with possible PTSD compared to four controls. Su et al. (2008) analysed mitochondrial gene expression in six PTSD brain samples and identified mitochondrial dysfunction in the dorsolateral prefrontal cortex in PTSD that may prove useful to improve diagnosis and treatment of PTSD. The rarity of post-mortem studies on PTSD is a direct reflection of the worldwide serious shortage of human psychiatric brain tissue available for post-mortem research.

## 1. Brain Banks worldwide

Only a limited number of Brain Banks have collected post-mortem psychiatric brains for research purposes of which only some include also PTSD cases (Deep-Soboslay et al., 2011). Therefore, several initiatives to increase numbers of PTSS cases were launched worldwide, such as the McLean’s Harvard Brain Tissue Resource Center, the Leahy-Friedman National PTSD Brain Bank in Boston (Thompson, 2015), but also our initiative in the Netherlands.

Unfortunately, in many cases Brain Banks lack clinical information of the donors, specifically detailed information on the course of the disease and drug use that was collected in a standardized manner. Such information is crucial for interpreting findings in the brain. Consequently, there is an urgent need for well-documented and high quality human brain tissue from patients with psychiatric disorders.

## 2. Netherlands Brain Bank for psychiatry

The Netherlands Brain Bank (NBB) launched a prospective donor programme to recruit brain donors with psychiatric diseases in 2012: the Netherlands Brain Bank for Psychiatry (NBB-Psy) (Rademaker, De Lange, & Palmen, in press). NBB-Psy aims to establish a resource of brain tissue of seven major psychiatric disorders: PTSD, major depressive disorder, schizophrenia, bipolar disorder, obsessive-compulsive disorder, autism spectrum disorder, and attention-deficit hyperactivity disorder. To accomplish this, together with research groups of five Dutch Academic Medical Centers we developed a strategy based on two strong assets in the Netherlands. First, the NBB is one of the world’s leading Brain Banks and well known for its rapid fresh dissection protocols (4–10 hours after death). Second, the availability of several large and clinically characterized research cohorts of psychiatric patients, including relatives and controls. Participants of these cohorts were asked prospectively to register as brain donors at the NBB. Additionally, we approached patient organizations, we asked long-stay and specialized clinics to exhibit our brochures in the waiting areas, and we launched a national campaign in which we made an appeal through radio, television and billboards to register as brain donor. We invited registered donors to cooperate with a psychiatric interview and complete annual medical questionnaires. State-of-the-art neuropathological diagnoses are performed for each donor. A full clinical and neuropathological report accompanies each entry of available tissue at the NBB. Paraffin and frozen tissue blocks from standard regions from both hemispheres are available for researchers. Furthermore, the NBB is the only Brain Bank in the world that enriches the material by acute isolation of pure glial cells which are increasingly important as they are more and more recognized as key players in the pathophysiology of psychiatric disorders (Chung, Welsh, Barres, & Stevens, 2015; Elsayed & Magistretti, 2015; Mizee, van der Poel, & Huitinga, in press). One hallmark enrichment strategy of NBB-Psy is to isolate, characterize, and store microglia. A similar project is underway for the isolation of astrocytes. In addition, pluripotent stem cell lines that have been generated from donor fibroblasts are available, enabling researchers to study disorder-specific glial cells and neurons.

## 3. Worldwide open access

Since NBB-Psy started, the number of registered donors with a psychiatric disorder at the NBB has risen from 312 (most of which were patients with major depressive disorder) in the year 2010 to 1187 in 2017, of which 146 PTSD patients. The NBB guarantees worldwide open access to biomaterials and data. Any researcher affiliated with a research institute can apply. This resource of brain tissue allows researchers to address questions such as: (1) Are the numbers of neuronal or glial cells altered in specific brain regions? (2) Are the morphology and phenotype of these cells altered in specific brain regions? (3) Is there a change in neuronal function? (4) Are there synaptic abnormalities? (5) Which molecular pathways are affected? Addressing these questions is a crucial step towards advanced understanding of PTSD and other psychiatric disorders. The ultimate goal of NBB-Psy is to facilitate the development of better treatment strategies and a better quality of life for people with psychiatric disorders and their relatives.
